
JOURNAL INFORMATION
==============================
NLM Title Abbreviation: Sci Technol Adv Mater
Journal ID: Sci Technol Adv Mater
NLM Journal ID: 101614420
Journal ID: tsta20

Science and Technology of Advanced Materials

ISSN: 1468-6996
EISSN: 1878-5514
Publisher: Taylor & Francis

ARTICLE INFORMATION
==============================
PMCID: PMC5099670
PMID: 27877709
DOI: 10.1088/1468-6996/15/5/050301
Article ID: 11745904
Article version: 1
Subjects: Preface, Focus on Complex Metallic Phases

Preface: Focus on complex metallic phases

Tsai An-Pang a
Grin Yuri b
de Boissieu Marc c
a Institute of Multidisciplinary Research for Advanced Materials , Tohoku University , Sendai 980-8577 , Japan
b Max-Planck-Institut for Chemical Physics of Solids , 01187 Dresden Germany
c Sciences de l'Ingéniére , des Matériaux et des Procédés , Grenoble INP, CNRS, 28402 cedex France
Publication date: 2014 Oct
Electronic publication date: 2014 Sep 8
Volume: 15
Issue: 5
Electronic Location ID: 050301
Received 2014 Jul 7
Copyright: © 2014 National Institute for Materials Science
Copyright year: 2014
Copyright holder: National Institute for Materials Science
License: Content from this work may be used under the terms of the Creative Commons Attribution 3.0 licence. Any further distribution of this work must maintain attribution to the author(s) and the title of the work, journal citation and DOI.
License URL: https://creativecommons.org/licenses/by/3.0/

Reference count: 0
Page count: 1

==============================
OPEN ACCESS
IOP Publishing | National Institute for Materials Science

Science and Technology of Advanced Materials

Sci. Technol. Adv. Mater. **15** (2014) 050301 (1pp)

doi:10.1088/1468-6996/15/5/050301

### Preface

### Focus on complex metallic phases

**Guest Editors**

**An-Pang Tsai[1], Yuri Grin[2] and Marc de Boissieu[3]**

[1] *Institute of Multidisciplinary Research for Advanced Materials, Tohoku University, Sendai 980-8577, Japan*
[2] *Max-Planck-Institut for Chemical Physics of Solids, 01187 Dresden, Germany*
[3] *Sciences de l'Ingéniérie, des Matériaux et des Procédés, Grenoble INP, CNRS, 28402 cedex, France*
E-mail: aptsai@tagen.tohoku.ac.jp

The discovery of quasicrystal in 1982 has facilitated a revolution in crystallography and led to a paradigm shift in our understanding of the structure of ordered solids. The importance of the discovery was demonstrated by the fact that Professor Shechtman was awarded the 2011 Nobel Prize in Chemistry. More than 30 years since the discovery, the field has stimulated an enormous scientific activity ranging from pure mathematics to practical materials. The study of quasicrystal has spread to a new field, which covers complex metallic phases and/or quasicrystal approximants. In fact, it was only very recently that a better understanding of structure of quasicrystal has been attained and some applications in the field of complex metallic alloys have been tackled. The purpose of this issue is to present the current development in the basic and application-driven research on complex metallic phases, including the quasicrystals and their approximants.

The issue contains 12 papers altogether, covering the new challenge of materials design for quasicrystals in several directions, such as structural materials, catalysts, thermoelectric materials and thermal rectification, new developments for related intermetallic compounds and the latest development on physical properties and surfaces. The papers are all written by excellent scientists in the field. Some of them are review articles, which will help readers understand the background of quasicrystals and the rest are original papers, which will show the state of the art of the field. We hope this issue will be helpful for scientists who want to learn the basic of quasicrystals or who want to know the recent progress on complex metallic phases in terms of materials science. Last but not the least, we are grateful to all authors for their contribution on this issue.

Finally, we would like to thank Drs T Tanaka, T Hatano and T Yoshida at the editorial office of STAM for their generous help in progressing the review process.

Content from this work may be used under the terms of the Creative Commons Attribution 3.0 licence. Any further distribution of this work must maintain attribution to the author(s) and the title of the work, journal citation and DOI.

© 2014 National Institute for Materials Science
